# Tirzepatide-associated interstitial kidney injury

**DOI:** 10.1210/jcemcr/luag125

**Published:** 2026-05-05

**Authors:** Mohamed Eldib, Vaidehi Mendpara, Leal Herlitz, Paloma Rodriguez Alvarez, Jagmeet Dhingra, Leila Zeinab Khan

**Affiliations:** Endocrinology and Metabolism Institute, Cleveland Clinic, Cleveland, OH 44195, USA; Department of Internal Medicine, Cleveland Clinic, Cleveland, OH 44195, USA; Department of Anatomic Pathology, Cleveland Clinic, Cleveland, OH 44195, USA; Endocrinology and Metabolism Institute, Cleveland Clinic, Cleveland, OH 44195, USA; Department of Kidney Medicine, Cleveland Clinic Foundation, Cleveland, OH 44195, USA; Endocrinology and Metabolism Institute, Cleveland Clinic, Cleveland, OH 44195, USA

**Keywords:** tirzepatide, glucagon-like peptide 1 receptor agonists, drug-induced kidney injury, tubulointerstitial nephritis, interstitial kidney injury, type 2 diabetes mellitus

## Abstract

We report a case of interstitial nephritis, likely secondary to tirzepatide. A 66-year-old man with type 2 diabetes, stage 3b chronic kidney disease, and other metabolic comorbidities experienced a progressive decline in renal function. Serum creatinine rose from 1.1 mg/dL (SI: 97.2 µmol/L) to 2.11 mg/dL (SI: 186.5 µmol/L) (reference range, 0.6-1.2 mg/dL [SI: 53-106 µmol/L]); estimated glomerular filtration rate (eGFR) fell from 68 to 34 mL/min/1.73 m^2^ over 8 months while receiving escalating doses of tirzepatide. Despite cessation of other medications and supportive care, renal dysfunction persisted. Kidney biopsy revealed chronic active tubulointerstitial nephritis with eosinophilic infiltrates and fibrosis implicating tirzepatide as the likely cause. Discontinuation of tirzepatide and initiation of prednisone resulted in significant improvement (serum creatinine measured 1.68 mg/dL (SI: 148.5 µmol/L) at 1 month and stabilized at 1.78 mg/dL (SI: 157.3 µmol/L); eGFR improved from 34 mL/min/1.73 m^2^ to 42 mL/min/1.73 m^2^ by 3 months). This case highlights a rare, biopsy-proven adverse renal effect of tirzepatide and underscores the importance of considering interstitial nephritis in patients with unexplained kidney injury on incretin mimetic therapy. Although glucagon-like peptide-1 receptor agonists benefits outweigh rare risks of nephrotoxicity, regular monitoring of creatinine monitoring is advised.

## Introduction

Glucagon-like peptide-1 receptor agonists (GLP-1 RAs) are widely used for type 2 diabetes (T2D) and obesity. They benefit glycemic control, provide cardiovascular risk reduction, and provide renal benefits. There have been multiple reports of acute kidney injury (AKI) associated with GLP-1 receptor agonists, leading to a Food and Drug Administration (FDA) warning and precautions [1 ]. While most reported cases lacked biopsy confirmation, a few biopsy-proven instances of interstitial nephritis have been described with liraglutide, dulaglutide, and semaglutide [2-6]. Tirzepatide, a dual GLP-1 and glucose-dependent insulinotropic polypeptide (GIP) agonist, is increasingly prescribed, yet biopsy-confirmed interstitial nephritis has not previously been reported with this agent. This case represents the first report of tirzepatide-induced interstitial nephritis confirmed by renal biopsy, thus highlighting a need for increased vigilance and further study of renal adverse effects in this drug class.

## Case presentation

**Figure 1:**
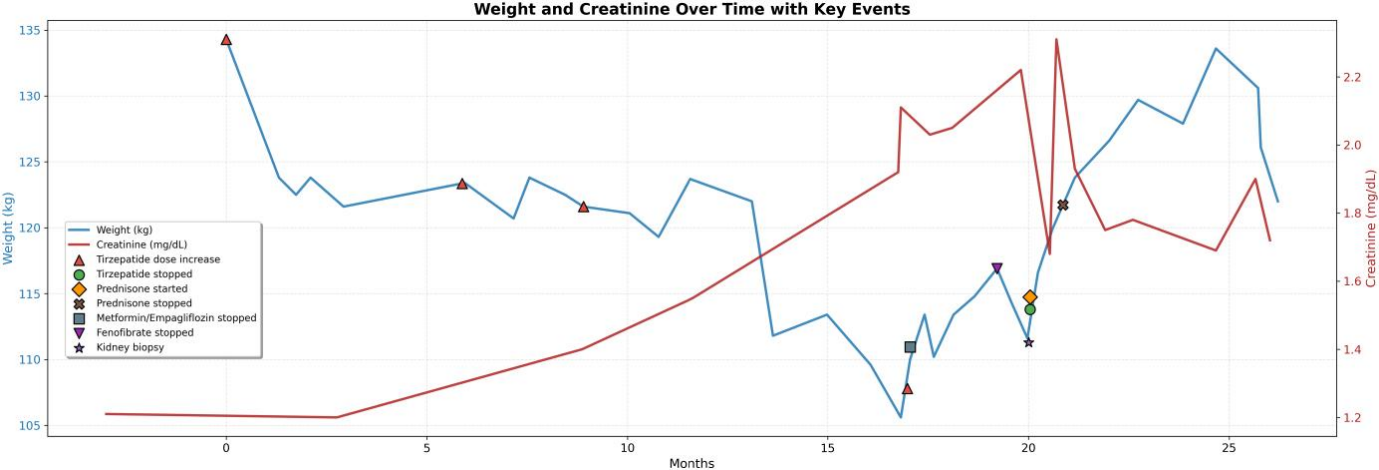
Weight and creatinine over time with key clinical events. The blue line represents body weight (kg) and the red line represents serum creatinine (mg/dL). Red upward triangles indicate tirzepatide dose increases. Green circles denote when tirzepatide was stopped. Orange diamonds mark when prednisone was started, and X symbols indicate when prednisone was stopped. Blue squares represent discontinuation of metformin/empagliflozin. Purple downward triangles indicate fenofibrate discontinuation. Purple stars mark the timing of the kidney biopsy.

A 66-year-old man had T2D, hypertension, stage 3b chronic kidney disease (CKD) (likely secondary to obesity and diabetic nephropathy), hyperlipidemia, osteoarthritis, and gastroesophageal reflux disease. He was receiving metformin (1000 mg twice daily), empagliflozin (10 mg daily), tirzepatide (10 mg weekly), lisinopril (10 mg daily), fenofibrate (160 mg daily), and pantoprazole (20 mg daily). He was started on tirzepatide to improve glycemic control and his weight. He received tirzepatide 5 mg weekly for 6 months, followed by 7.5 mg for another 6 months. Over the course of the year, he experienced a weight reduction of 14 kg. His weight decreased from 136 to 122 kg. During this period, his hemoglobin A1c (HbA1c) decreased from 6.9% (SI: 52 mmol/mol) to 5.5% (SI: 37 mmol/mol) (reference range, 4.0-5.6% [SI: 20-38 mmol/mol]). After this first year, the tirzepatide dose was further increased to 10 mg and maintained for 8 months. During an outpatient visit, serum creatinine was found to be elevated during a mild self-limited diarrheal episode without any significant dehydration. A review of the patient's kidney function during the prior 8 months revealed a gradual decline in kidney function with serum creatinine increasing from 1.1 mg/dL (SI: 97.2 µmol/L) (reference range, 0.6-1.2 mg/dL [SI: 53-106 µmol/L]) to 2.11 mg/dL (SI: 186.5 µmol/L) and estimated glomerular filtration rate (eGFR) decreasing from 68 to 34 mL/min/1.73 m^2^ (reference range, ≥90 mL/min/1.73 m^2^). The clinical course is summarized in Fig. 1.

## Diagnostic assessment

**Figure 2:**
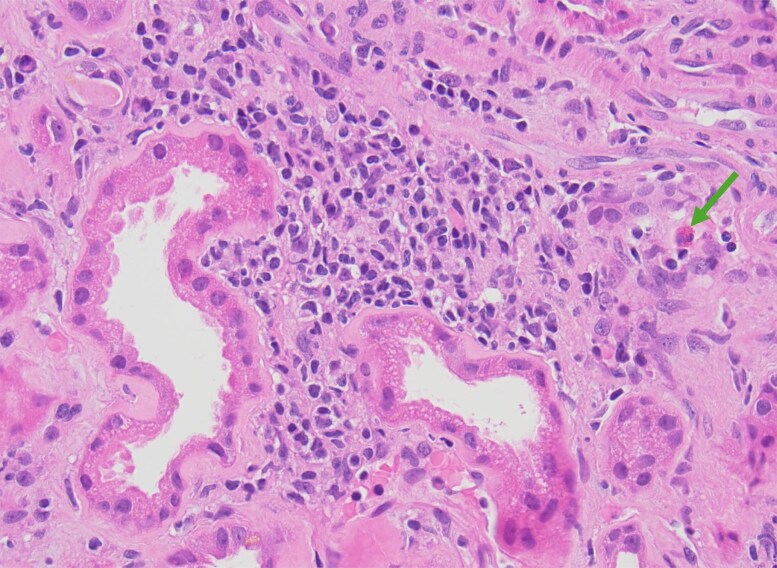
Interstitial inflammation by a predominantly mononuclear infiltrate with an occasional eosinophil (arrow). H&E 200× magnification.

**Figure 3:**
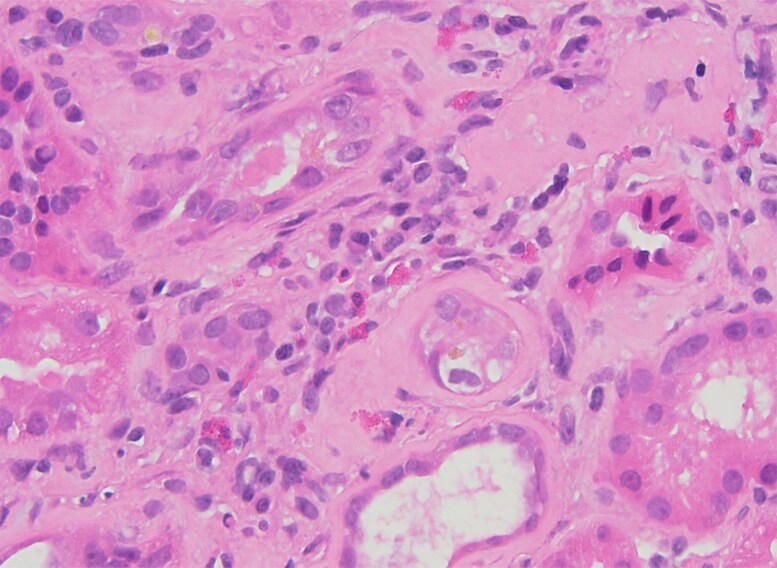
High power of the patchy inflammation showing multiple eosinophils in the interstitium (H&E 400×).

Due to this decrease in renal function, the metformin, empagliflozin and fenofibrate were promptly discontinued. Pantoprazole had already been self-discontinued by the patient, and tirzepatide was further increased to 12.5 mg to improve glycemic control. He then underwent an initial assessment by nephrology, which attributed the worsening renal function to dehydration in the context of underlying stage 3b CKD. Urinalysis was negative for hematuria and proteinuria. A 24-hour urine collection was not performed. Kidney ultrasound was unremarkable and revealed a normal parenchymal echogenicity. Despite the above interventions, the creatinine remained at 2.22 mg/dL (SI: 196.2 µmol/L) 3 months later. Subsequent kidney biopsy revealed chronic active tubulointerstitial nephritis with eosinophilic infiltrates and fibrosis (Figs. 2 and 3). Tirzepatide was identified by nephrology as the likely cause.

## Treatment

Once tirzepatide was identified as the underlying cause, it was immediately discontinued and the patient was started on oral prednisone 40 mg daily for 5 days, followed by a taper from 30 to 5 mg daily over one month.

## Outcome and follow-up

Tirzepatide discontinuation and treatment with oral prednisone led to significant improvement in renal function. Serum creatinine decreased to 1.68 mg/dL (SI: 148.5 µmol/L) by month one and stabilized at 1.78 mg/dL (SI: 157.3 µmol/L) with an eGFR of 42 mL/min/1.73 m^2^ by month 3.

## Discussion

AKI denotes a rapid decline in renal filtration capacity typically reflected by a reduced glomerular filtration rate (GFR) and elevations in blood urea nitrogen and serum creatinine [7]. The etiologies of AKI are broadly categorized as prerenal, intrarenal, or postrenal, with intrarenal causes such as acute interstitial nephritis (AIN) accounting for nearly half of all intrinsic cases [8]. Drug-induced AIN remains a recognized yet frequently underdiagnosed source of kidney injury. It is historically associated with antibiotics, non-steroidal anti-inflammatory drugs, and proton-pump inhibitors. Increasingly, however, novel metabolic therapies have been implicated [9, 10].

In our patient, the temporal relationship between escalating tirzepatide dose and progressive renal dysfunction, absence of other nephrotoxins, and biopsy findings of chronic active tubulointerstitial nephritis with eosinophilic infiltration support tirzepatide-associated AIN. Although transient dehydration during a mild self-limited diarrheal illness could have added a prerenal component, the persistence of renal impairment despite supportive measures and medication withdrawal suggests a direct immune-mediated mechanism. The lack of fever, rash, or eosinophilia parallels previous descriptions of subclinical, delayed T-cell–mediated hypersensitivity patterns observed in drug-induced AIN [10].

AKI due to volume depletion related to gastrointestinal side effects remains the most frequent renal adverse effect of glucagon-like peptide-1 receptor agonists (GLP-1RAs) [11, 12]. However, biopsy-proven AIN has been described with several agents in this class, including liraglutide [2, 3], dulaglutide [4], and semaglutide [13, 14]. Our case extends this complication to tirzepatide, a dual glucose-dependent insulinotropic polypeptide (GIP) and GLP-1 receptor agonist. Approved by the U.S. FDA in 2022 for T2D and subsequently for obesity and more recently for management of obstructive sleep apnea [15-17], tirzepatide acts through complementary incretin pathways that enhance glycemic and weight-reducing efficacy via biased cyclic adenosine monophosphate signaling and prolonged receptor activity [18-21]. These effects collectively contribute to improved renal hemodynamics, yet rare paradoxical immune reactions such as AIN underscore the complex interplay between pharmacologic benefit and hypersensitivity. Clinical studies, including a recent publication have demonstrated substantial HbA1c and body weight reductions, however, gastrointestinal adverse events are common and occur in approximately 10% of patients [22]. These side effects are generally transient and dose dependent [23]. While these symptoms can occasionally precipitate dehydration-related prerenal AKI, the drug's overall renal profile has been considered protective rather than toxic [24, 25].

In the SURPASS-4 trial, tirzepatide slowed the decline in eGFR and reduced albuminuria relative to insulin glargine, yielding a 42% lower risk of composite kidney outcomes including ≥40% eGFR reduction, macroalbuminuria, or kidney failure [26, 27]. The early eGFR dip followed by stabilization mirrored the trajectory seen with nephroprotective therapies such as sodium–glucose cotransporter 2 inhibitors and renin–angiotensin–aldosterone system blockers. Mechanistically, incretin receptor activation in renal tubular and vascular cells enhances natriuresis, nitric oxide–mediated vasodilation, and anti-inflammatory signaling, while GIP activity may promote adiponectin secretion and mitigate oxidative stress [28]. These collective data suggest that tirzepatide exerts kidney-protective effects making the present biopsy-confirmed AIN a notable paradox.

Drug-induced AIN, a delayed T-cell–mediated immune response [26], may lead to interstitial fibrosis if untreated; however, early corticosteroid therapy, as shown in prior studies [28], facilitates renal recovery. In our patient, timely discontinuation of tirzepatide and corticosteroid initiation led to partial renal recovery. This is consistent with prior observations that early immunosuppression favors renal recuperation.

This case emphasizes the need for clinical vigilance when evaluating unexplained renal dysfunction in patients receiving incretin-based therapy. Despite this rare adverse event, the overall benefits of GLP-1–based therapies continue to outweigh the risks; nonetheless, regular monitoring of renal function, including serum creatinine and eGFR, is recommended for all patients receiving these agents. Although tirzepatide provides robust metabolic and renal benefits in clinical trials, clinicians should remain aware of rare immune-mediated renal events. Early recognition, cessation of the offending agent, and prompt corticosteroid therapy are critical to preventing irreversible injury. Continued pharmacovigilance and mechanistic studies are warranted to clarify host–drug interactions and immunopathogenic triggers. Additional research is needed to fully understand this emerging adverse effect.

## Learning points

- Tirzepatide, a dual GIP and GLP-1 receptor agonist, is generally kidney-protective but can, in rare cases, trigger biopsy-proven AIN.
- Drug-induced AIN may present without classical allergic features and should be suspected when renal function declines after dose escalation of incretin therapies.
- Prompt drug discontinuation and early corticosteroid therapy are key to renal recovery and prevention of CKD progression.
- With the expanding use of tirzepatide for diabetes and obesity, clinician vigilance and post-marketing pharmacovigilance are essential to identify and characterize rare immune-mediated renal complications.

## Contributors

All authors made individual contributions to authorship. L.Z.K. and J.D. were responsible for diagnosis and management of the patient. M.E., V.M., and P.R.A. were involved in the conception and design of the work, the acquisition, analysis, and interpretation of data, and in manuscript writing. L.H. was responsible for the histopathology section and preparation of histology images. All authors reviewed and approved the final draft.

## Data Availability

Data sharing is not applicable to this article as no datasets were generated or analyzed during the current study.

## Abbreviations

AIN: acute interstitial nephritis
AKI: acute kidney injury
CKD: chronic kidney disease
eGFR: estimated glomerular filtration rate
FDA: Food and Drug Administration
GFR: glomerular filtration rate
GIP: glucose-dependent insulinotropic polypeptide
GLP-1RA: glucagon-like peptide-1 receptor agonist
